# Changes in Lipid Profile, Body Weight Variables and Cardiovascular Risk in Obese Patients Undergoing One-Anastomosis Gastric Bypass

**DOI:** 10.3390/ijerph17165858

**Published:** 2020-08-13

**Authors:** Jose-Maria Jiménez, Miguel-Angel Carbajo, María López, María-José Cao, Jaime Rúiz-Tovar, Sara García, María-José Castro

**Affiliations:** 1 Center of Excellence for the Study and Treatment of Diabetes and Obesity, 47004 Valladolid, Spain; doctormacarbajo@gmail.com (M.-A.C.); jruiztovar@gmail.com (J.R.-T.); mariajose.castro@uva.es (M.-J.C.); 2 Nursing Faculty, University of Valladolid, 47005 Valladolid, Spain; mjcao@enf.uva.es (M.-J.C.); sara.garcia.villanueva@uva.es (S.G.); 3 Endocrinology and Clinical Nutrition Research Center (ECNRC), University of Valladolid, 47005 Valladolid, Spain

**Keywords:** morbid obesity, lipid profile, weight loss, one-anastomosis gastric bypass, cardiovascular risk

## Abstract

Morbid obesity has a direct impact on the development of cardiovascular disease. One-anastomosis gastric bypass (OAGB) is an effective surgical technique for the control of body weight and the reduction of cardiovascular risk. This work examines the change in weight loss, lipid profile and cardiovascular risk in 100 patients (71 women, 29 men), mean age 42.61 ± 11.33 years at 3, 6, 9, 12, 18 and 24 months after OAGB. At 24 months post-surgery, mean body weight was significantly reduced compared to pre-operative values (116.75 ± 22.19 kg vs. 69.66 ± 13.07 kg), as were mean total cholesterol (201.86 ± 44.60 mg/dL vs. 172.99 ± 32.26 mg/dL), LDL (Low-Density Lipoprotein) cholesterol (126.90 ± 39.81 mg/dL vs. 96.28 ± 26.99 mg/dL), triglycerides (138.05 ± 78.45 mg/dL vs. 76.04 ± 30.34 mg/dL) and cardiovascular risk (total cholesterol/HDL (High-Density Lipoprotein) cholesterol: 4.32 ± 1.24 vs. 2.93 ± 0.71), while the mean HDL cholesterol concentration was significantly higher (49.09 ± 14.16 mg/dL vs. 61.98 ± 14.86 mg/dL) (all *p* < 0.001). In conclusion, OAGB surgery led to significant reductions in body weight, a significant improvement in the lipid profile, and a reduction in cardiovascular risk.

## 1. Introduction

The World Health Organization (WHO) defines obesity as abnormal or excessive fat accumulation that may affect health [1]. Certainly, the condition increases the risk of developing cardiovascular disease, diabetes, musculoskeletal disorders and cancer in a manner proportional to the increase in body mass index (BMI). Worldwide, more than 1.9 billion people over 18 years of age were overweight in 2016; 650 million of them were obese [1]. According to the Global Burden of Disease Project, in 2015 excess bodyweight accounted for a global 4 million deaths and 120 million disability adjusted life years. Almost 70% of deaths related to high BMI were caused by cardiovascular disease, and over 60% of those deaths were among obese people [2].

Obesity is associated with high plasma concentrations of triglycerides, cholesterol and LDL (Low-Density Lipoprotein) cholesterol, and low concentrations of HDL (High-Density Lipoprotein) cholesterol. However, overweight, obesity and dyslipidemia are all modifiable risk factors of cardiovascular disease [3,4]. The treatment of choice for obesity depends upon the type of obesity and the clinical features of each patient, but generally begins with modification of the diet, health education, and medication [5,6,7]. Surgical treatment is, however, an option for some patients [8,9]. The main procedures include gastric bypass, a technique that reduces nutrient absorption (performed in 46.3% of cases), and sleeve gastrectomy, which restricts nutrient absorption (performed in 43.6% of cases) [10]. These techniques are associated with a 15–30% total weight loss (TWL) [11], improved weight control and a better lipid profile [12,13]. Indeed, even a 5–10% weight loss can lead to improved lipid metabolism [14], increasing the HDL cholesterol concentration and lowering those of triglycerides, total cholesterol and LDL cholesterol [15].

Compared to the above type of surgery, one-anastomosis gastric bypass (OAGB) is a more modern, less invasive (laparoscopic) mixed nutrient absorption reducing-restricting surgical technique, with the potential to provide similar results. The aim of the present work was to examine the change over a 24-month period in weight loss, lipid profile and cardiovascular risk in patients who had undergone OAGB.

## 2. Materials and Methods

### 2.1. Study Design

This descriptive study with prospective collection of data was performed at the Centre of Excellence for the Study and Treatment of Obesity and Diabetes in Valladolid (Spain). The study subjects were 100 Caucasian patients (71 women, 29 men) who underwent OAGB surgery between January and December of 2010. All fulfilled the indication criteria for bariatric-metabolic surgery approved by the International Federation for the Surgery of Obesity (IFSO), i.e., a BMI of >40 kg/m^2^, or ≥30–35 kg/m^2^ plus a progressive deterioration of glycemic control, plus important but inadequately managed comorbidities [16].

Pre-surgery examination included the recording of patient sex and age, anamnesis, blood typing, anthropometric measurements, psychological and radiological evaluations, and cardiorespiratory function testing. Surgery was performed (always by the same surgical team) under general anesthesia and using a previously described laparoscopic technique [17]. Postoperative care included providing assistance to patients in their adaptation to new eating habits and increased physical activity. Following the recommendations of the American Society of Metabolic and Bariatric Surgeons (ASMBS) [18], the following variables were monitored: (1) changes in patient body weight, BMI [weight (Kg)/height^2^ (m)], percentage excess weight loss (%EWL = [pre-operative weight − current weight/pre-operative weight − ideal weight] × 100 [note: ideal body weight was defined by the weight corresponding to a BMI of 25 kg/m^2^]), percentage excess body mass index loss (%EBMIL = [pre-operative BMI − current BMI/pre-operative BMI − 25] × 100]), and percentage total weight loss (%TWL = [pre-operative weight – current weight]/[pre-operative weight] × 100], (2) lipid profile, i.e., total cholesterol, HDL cholesterol, LDL cholesterol and triglycerides, and (3) cardiovascular risk (total cholesterol/HDL cholesterol).

Data were recorded at 3, 6, 9, 12, 18 and 24 months post-surgery. To determine the lipid profile, venous blood was collected after an overnight fasting. Lipid profile (high density lipoprotein, total cholesterol and triglycerides) were determined from the serum, following standard operating procedures. Commercial reagents are used, which include the enzymes and substrates necessary for the quantification of all forms of cholesterol present in the serum.

According to the clinical guidelines of the National Cholesterol Education Program (NCEP) Adult Treatment Panel III (ATP-III) for the diagnosis and treatment of dyslipidemia [19], a total cholesterol of <200 mg/dL was deemed normal, 200–239 mg/dL as borderline high, and >240 mg/dL as high. An LDL cholesterol concentration of <100 mg/dL was deemed optimal, 100–129 mg/dL as near optimal, 130–159 mg/dL as borderline high, 160–189 mg/dL as high, and ≥190 mg/dL as very high. The HDL cholesterol concentration was deemed low at <40 mg/dL, while >60 mg/dL was considered high. A triglyceride concentration of <150 mg/dL was deemed normal, 150–199 mg/dL as borderline high, 200–499 mg/dL as high, and ≥500 mg/dL as very high. In addition, a cardiovascular risk score (calculated as described above) of ≤3.27 was deemed ½ the average risk, up to 4.44 as the average risk, then up to 7.05 as twice the average, and then up to 11.04 as three times the average. Class I obesity was defined as BMI ≥ 30 ≤ 34.9 kg/m^2^, class II as BMI ≥ 35 ≤ 39.9 kg/m^2^, class III (morbid obesity) as BMI ≥ 40 ≤ 49.9 kg/m^2^, and class IV (extreme obesity) as BMI ≥ 50 kg/m^2^.

### 2.2. Statistical Analysis.

All calculations were performed using SPSS v.5.0 software (SPSS Inc.; Chicago, IL, USA). Quantitative variables were defined by mean and standard deviation when they followed a Gaussian distribution. Qualitative variables were defined by percentage. The paired t test or Wilcoxon rank test was used to study the differences between means over time, and ANOVA or the Kruskal-Wallis test for comparing changes in variables with more than two categories. Significance was set at *p* < 0.05.

### 2.3. Ethics Statement

This study was approved by the University of Valladolid Institutional Review Board and the Ethics Committee of the Faculty of Nursing (ID code: 2019/JMJP39). Informed written consent was obtained from all subjects. In the case of underage patients, the informed consent was completed by the parents or guardians and always informing and having the approval of the minor.

## 3. Results

The mean age of the patients at baseline was 42.61 ± 11.33 years (range 13–65 years); women: 42.56 ± 11.33 years, men: 42.72 ± 12.25 years. The mean pre-operative BMI was 42.61± 6.66 kg/m^2^ (range 30–58.98 kg/m^2^), with no significant difference between women and men (42.32 ± 6.75 kg/m^2^ vs. 43.31 ± 6.49 kg/m^2^). Before surgery, 10% of the patients were class I obese (7% women, 3% men), 33% fell into class II (26% women, 7% men), 42% into class III or morbid obesity (28% women, 14% men), and 15% into class IV or extreme obesity (10% women, 5% men).

### 3.1. Weight Loss

Table 1 shows the change in weight loss variables of the patients who underwent OAGB. Significant weight loss was observed at all post-surgery check points with respect to initial body weight (*p* < 0.001). For the population as a whole, the lowest mean weight was reached 12 months after surgery (68.56 ± 13.10 kg); in men this was reached at 12 months (78.96 ± 12.94 kg), and in women at 18 months (63.57 ± 10.96 kg). At each check point a higher average body weight was observed for the men (*p* < 0.001).

Mean BMI also fell over time, reaching its lowest at 12 months post-surgery (25.08 ± 3.59 kg/m^2^). After this time the BMI increased, although the values always remained significantly lower than the pre-operative BMI (*p* < 0.001). No difference was seen between the BMI of the men and women before surgery (43.31 ± 6.49 kg/m^2^ vs. 42.32 ± 6.75 kg/m^2^), but the mean BMI of the men was higher than that of the women at the 18-month (26.90 ± 3.31 kg/m^2^ vs. 24.46 ± 3.41 kg/m^2^) and 24-month check points (26.70 ± 3.05 kg/m^2^ vs. 24.77 ± 3.32 kg/m^2^) (*p* < 0.05). After 24 months, a normal BMI (≥ 18.5 ≤ 24.9 kg/m^2^) was attained by 48% of patients; 43% were still overweight (BMI ≥ 25 ≤ 29.9 kg/m^2^), just 8% had class I obesity (BMI ≥ 30 ≤ 34.9 kg/m^2^), and none was morbidly or extremely obese.

The %EWL increased progressively over time, with the highest value seen at 12 months post-surgery (89.70% ± 16.57%, *p* < 0.001 compared to the first possible checkpoint at 3 months). At the 3-month check point no significant difference was seen between the men and women in terms of %EWL (70.68% ± 15.13% vs. 65.30% ± 18.25%). However, the women returned a significantly higher %EWL value than the men at 12, 18 and 24 months (*p* < 0.05).

The %EBMIL followed the same trend as BMI. Women obtained better results than men from the 6-month check point onwards, reaching significance at the 12-, 18- and 24-month check points (*p* < 0.05).

The %TWL increased by 6 months, with the highest values reached at 12 months post-surgery (41.30% ± 7.42%). The women returned better results than the men from 6 months onwards, reaching significance at 18 and 24 months (*p* < 0.05).

### 3.2. Change in Lipid Profile

Before surgery, 68% of the patients (49 women and 19 men) had dyslipidemia; 36.76% of them were following hypolipidemic treatments. Table 2 shows the change in the lipid profile following surgery. Total cholesterol and LDL cholesterol concentrations were significantly lower at every check point compared to pre-operative values.

**Figure 1:**
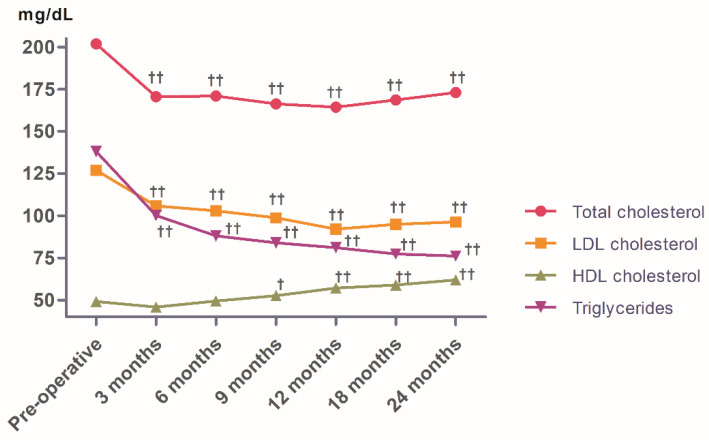
Evolution of changes in the lipid profile (total cholesterol, LDL cholesterol, HDL cholesterol, triglycerides) after bariatric surgery. ^†^ *p* < 0.05 or ^††^ *p* < 0.001 compared to the pre-operative check point. Results were expressed with mean. Units of measurement: mg/dL

Figure 1 shows the evolution of changes in the lipid profile, the lowest total cholesterol and LDL cholesterol concentrations were detected at 12 months (164.38 ± 28.25 mg/dL and 91.99 ± 24.75 mg/dL respectively). The women returned a mean total cholesterol level higher than that of the men at every check point (*p* < 0.05). No significant differences were seen between men and women in terms of LDL cholesterol. HDL cholesterol increased significantly from the 6-month check point onwards, reaching its highest value 24 months after surgery (61.98 ± 14.86 mg/dL). The women returned a higher mean HDL cholesterol value than the men at every check point. Both sexes returned the highest values at 24 months (men: 56.14 ± 16.17 mg/dL. women: 64.35 ± 13.72 mg/dL).

Triglycerides decreased progressively over time, with the lowest values (taking both sexes together) recorded 24 months after surgery (76.04 ± 30.34 mg/dL). A progressive reduction was also seen for both sexes, with the lowest mean value for women reached at 24 months (73.87 ± 27.79 mg/dL), and the lowest for the men reached 18 months (79.31 ± 29.52 md/dL). At 24 months, no patients required lipid-lowering medication, 83.51% had a normal total cholesterol concentration (i.e., ≤200 mg/dL), 58.63% had a normal LDL cholesterol concentration (<100 mg/dL), 97.90% had a normal triglyceride concentration (150 mg/dL), 100% had an HDL cholesterol concentration of >40 mg/dl, and 54.44% had an HDL cholesterol concentration of >60 mg/dL.

**Figure 2:**
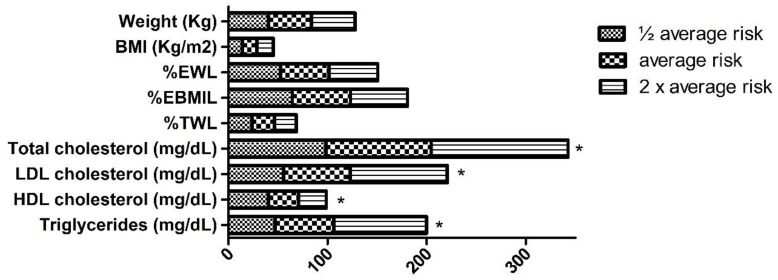
Change in weight, lipid profile and cardiovascular risk (according to ASBMS criteria) at 24 months post-surgery. * *p* < 0.001 when comparing cardiovascular risk groups.

Table 2 shows the change in cardiovascular risk (as reflected by total cholesterol/HDL cholesterol). The fall was notable at every check point compared to pre-operative values. According to the ASMBS classification, the percentage of patients at ½ the average risk increased from a pre-operative 47.1% to 88% at 24 months post-surgery; the percentage of patients at average risk decreased from 50% to 12% over the same period; and the percentage at twice the average risk fell from 2.9% to 0%. Figure 2 shows that, at 24 months, those patients at higher post-operative cardiovascular risk had a greater body weight and BMI, and lower %EWL, %EBMIL and %TWL values (*p* < 0.001).

Significant differences were found between the patients of the different cardiovascular risk groups in terms of their lipid profile variables (*p* < 0.001); those at higher risk had higher total cholesterol, LDL cholesterol and triglyceride concentrations, a higher total cholesterol/HDL cholesterol ratio, and a lower HDL cholesterol concentration (Figure 2).

## 4. Discussion

At 24 months after OAGB, the patients had lower body weights and better lipid profiles, reducing their risk of developing cardiovascular disease. Indeed, a significant weight loss was recorded at every post-surgery check point compared to pre-operative values.

In agreement with the present results, Chakhtoura et al. [20] reported the greatest weight loss (89.8 ± 18.4 kg) to be seen at 12 months after mini-gastric bypass (a reduction-restriction technique), while authors report the greatest weight loss at 18 months with this technique (79.3 ± 14.4 kg) [21].

Weight loss after OAGB was substantial, demonstrating the impact of weight loss in the modification of the lipid profile on the study cohort previously published by the same surgical team [22].

Bariatric surgery has been shown effective for achieving weight loss, with the best result obtained when using surgical techniques that reduce nutrient absorption. In the present study, which involved a combined nutrient absorption reduction-restriction technique, higher %EWL values were returned than with the Roux-en-Y gastric bypass (RYGB), another combined nutrient reduction-restriction technique at 12 months (89.70% vs. 53.8%) and 24 months (88.10% vs. 60.7%) [23].

The present weight loss results at 24 months were also better than those obtained by Ooi et al. who performed nutrient restriction surgery involving adjustable gastric banding (AGB) (%EWL 88.10% vs. 48.7%; %TWL 39.87% vs. 18.4%) [14].

Lee et al. reported higher %EWL values at 5 years for patients who underwent RYGB (a reduction-restriction technique) compared to those underwent sleeve gastrectomy (a restriction technique) (69.6% vs. 68.7%) [24].

In the present patients, total cholesterol and LDL cholesterol concentrations were significantly lower than pre-operative values through until 24 months post-surgery (but with no further improvement after 12 months). Similarly, HDL cholesterol was significantly higher than pre-operative values at all check points. Other authors describe a significant reduction in total cholesterol, LDL cholesterol and triglycerides, plus an increase in HDL cholesterol, at 6 and 12 months after different types of bariatric surgery, with RYGB (reduction-restriction) returning the best results [9,15,25] though perhaps less impressive than those obtained with the present OAGB technique. It would be interesting to eventually compare longer term results.

In the present patients, the triglyceride concentration was significantly reduced at every check point compared to the pre-operative values. Other authors report the same at 12 months after sleeve gastrectomy (restriction), and even more so in patients treated by RYGB (reduction-restriction) [26].

The present work suffers from a number of limitations. The sample was not randomly selected from several centers; all the patients were consecutive and from the same center. Further, the follow-up time was limited to 24 months. Future work should involve patients from different centers, longer follow-up periods, and take into account the impact of other health factors influenced by lifestyle.

## 5. Conclusions

OAGB would appear to be an effective surgical technique for achieving weight loss; both patient weight and BMI become more normal (in the present work no subject remained morbidly or extremely obese at 6 months). In addition, total cholesterol, triglycerides and LDL cholesterol concentrations fall, and the HDL cholesterol concentration rises. This weight loss, and the improvement in the lipid profile experienced, reduce the cardiovascular risk to which patients are exposed.

## Figures and Tables

**Table 1:** Change in the examined weight loss variables for the population as a whole.

	Pre-Operative	3 Months	6 Months	9 Months	12 Months	18 Months	24 Months
**Weight (kg)**	116.75 ± 22.19	81.55 ^††^ ± 15.22	74.02 ^††^ ± 14.11	72.13 ^††^ ± 14.78	68.56 ^††^ ± 13.10	69.67 ^††^ ± 14.40	69.66 ^††^ ± 13.07
**BMI (kg/m^2^)**	42.61 ± 6.66	29.77 ^††^ ± 5.04	26.99 ^†^ ± 4.15	26.44 ^†^ ± 3.92	25.08 ^†^ ± 3.59	25.27 ^†^ ± 3.54	25.33 ^††^ ± 3.35
**%EWL**	-	66.86 ± 17.49	81.05 ^††^ ± 17.64	83.31 ^††^ ± 15.37	89.70 ^††^ ± 16.57	88.40 ^††^ ± 16.93	88.10 ^††^ ± 16.99
**%EBMIL**	-	78.72 ± 24.12	95.50 ^††^ ± 26.66	96.96 ^††^ ± 20.76	104.82 ^††^ ± 23.57	103.43 ^††^ ± 24.16	103.79 ^††^ ±25.89
**%TWL**	-	30.19 ± 6.57	36.65 ^††^ ± 6.16	38.86 ^††^ ± 6.98	41.30 ^††^ ± 7.42	40.60 ^††^ ± 7.44	39.87 ^††^ ± 7.30

^†^*p* < 0.05, ^††^ *p* < 0.001 compared to pre-operative value or that recorded at first possible check point.

**Table 2:** Change in lipid profile after surgery.

	Pre-Operative	3 Months	6 Months	9 Months	12 Months	18 Months	24 Months
**Total Cholesterol**	201.86 ± 44.60	170.60 ^††^ ± 35.9	171.01 ^††^ ± 32.65	166.34 ^††^ ± 29.48	164.38 ^††^ ± 28.25	168.62 ^††^ ± 28.59	172.99 ^††^ ± 32.26
**LDL Cholesterol**	126.90 ± 39.81	105.82 ^††^ ± 35.78	102.90 ^††^ ± 29.40	98.80 ^††^ ± 24.03	91.99 ^††^ ± 24.75	94.88 ^††^ ± 25.01	96.28 ^††^ ± 26.99
**HDL Cholesterol**	49.09 ± 14.16	45.86 ± 13.91	49.37 ± 12.05	52.56 ^†^ ± 11.25	57.08 ^††^ ± 13.69	58.85 ^††^ ± 13.62	61.98 ^††^ ± 14.86
**Triglycerides**	138.05 ± 78.45	100.09 ^††^ ± 31.03	88.05 ^††^ ± 34.49	83.94 ^††^ ± 32.76	81.06 ^††^ ± 31.58	77.35 ^††^ ± 29.54	76.04 ^††^ ± 30.34
**Cardiovascular Risk. (TC/HDL Ratio)**	4.32 ± 1.24	3.93 ^††^ ± 1.09	3.61 ^††^ ± 0.91	3.27 ^††^ ± 0.65	3.05 ^††^ ± 0.90	2.99 ^††^ ± 0.72	2.93 ^††^ ± 0.71

^†^*p* < 0.05, ^††^ *p* < 0.001 compared to the pre-operative check point.
